# Gut Microbiota in Patients with Postoperative Atrial Fibrillation Undergoing Off-Pump Coronary Bypass Graft Surgery

**DOI:** 10.3390/jcm12041493

**Published:** 2023-02-13

**Authors:** Yang Wang, Yuanchen He, Rui Li, Hui Jiang, Dengshun Tao, Keyan Zhao, Zongtao Yin, Jian Zhang, Huishan Wang

**Affiliations:** 1 Department of Cardiovascular Surgery, General Hospital of Northern Theater Command, No. 83, Wenhua Road, Shenhe District, Shenyang 110016, China; 2 Postgraduate Training Base of Northern Theater Command General Hospital, Dalian Medical University, No. 9, Lvshun Wesztern South Road, Lvshunkou District, Dalian 116044, China; 3 Postgraduate Training Base of Northern Theater Command General Hospital, China Medical University, No. 83, Wenhua Road, Shenhe District, Shenyang 110016, China

**Keywords:** gut microbiota, post-operative atrial fibrillation, 16S rRNA, coronary artery bypass grafting, dysbiosis, vitamin D

## Abstract

Background: Post-operative atrial fibrillation (POAF) is one of the most common complications of cardiac surgery. However, the underlying mechanism is not well understood. Alterations in the gut microbiota are associated with the development of atrial fibrillation (AF). The aim of this study was to explore the relationship between gut microbiota and POAF. Methods: Fecal samples were collected before surgery from 45 patients who underwent coronary artery bypass grafting with POAF and 90 matched patients without POAF (1:2). 16S rRNA sequencing was used to detect the microbiome profiles of 45 POAF patients and 89 matched patients (one sample in the no-POAF group was deleted owing to low quality after sequencing). Plasma 25-hydroxy vitamin D level was measured by ELISA. Results: Compared to the patients without POAF, gut microbiota composition was remarkably changed in the patients with POAF, with an increase in *Lachnospira*, *Acinetobacter*, *Veillonella* and *Aeromonas*, and a decrease in *Escherichia–Shigella*, *Klebsiella*, *Streptococcus*, *Brevundimonas* and *Citrobacter*. Furthermore, plasma 25-hydroxy vitamin D levels were decreased in POAF patients and negatively correlated with an abundance of *Lachnospira*. Conclusions: The gut microbiota composition between patients with and without POAF is significantly different, implying that gut microbiota may play a role in the pathogenesis of POAF. Further studies are needed to fully clarify the role of gut microbiota in the initiation of AF.

## 1. Introduction

Post-operative atrial fibrillation (POAF) is the most common complication of cardiac surgery [1,2,3]. POAF is associated with long-term ischemic stroke, heart failure and mortality [4]. POAF increases the risk of ischemic stroke, heart failure hospitalization and recurrent atrial fibrillation (AF) [4,5,6,7]. Therefore, decreasing POAF may be beneficial to prevent the long-term risk of adverse outcomes. Several factors facilitate POAF, such as electrical ion channels and the atrial interstitial and extracellular matrices in atrium substrate [8]. Some acute factors directly related to surgery can provoke the initiation of arrhythmia, while some factors reflecting a chronic and progressive process of remodeling or aging of the heart enhanced the perpetuating AF. Additionally, numerous studies have found that abnormalities of the left atrium and ventricles are responsible for POAF initiation [4,9] Vitamin D, as an antioxidant agent, has been widely reported to be associated with AF. Vitamin D is believed to decrease atrial remodeling through the renin–angiotensin–aldosterone system. However, the detailed mechanisms underlying POAF are largely unknown. Therefore, determining potential biomarkers before surgery may help in identifying patients at high risk of POAF.

Gut microbiota influence several processes in the body, in addition to digestion [10,11]. After the heart–gut axis concept was proposed, many studies suggested that the gut microbiota are involved in the development of cardiovascular diseases, such as hypertension and AF [12,13,14]. Gut microbiota are significantly altered in AF patients. Some distinctive and progressive alterations occur in the gut microbiota and metabolic profiles with the progression of AF [15]. Patients with AF have an altered gut microbial composition because of dietary habits [16]. Mechanistically, gut microbiota may impact cardiac arrhythmogenesis through oxidative stress, inflammation and atrial fibrosis [14,17,18,19]. Gut microbiota composition is also influenced by vitamin D status. Low levels of vitamin D are associated with increased LPS, which is involved in elevated inflammation: two triggers for POAF. Therefore, vitamin D status along with gut microbiota may co-affect AF initiation. However, the clinical significance of the gut microbiota in AF initiation needs to be further explored. To identify the gut microbiota that initiate the onset of AF, we investigated whether patterns of dysbiosis gut microbiota were associated with POAF in patients who underwent isolated coronary artery bypass grafting (CABG).

## 2. Methods

### 2.1. Study Cohort

Fecal samples were collected before surgery from the Department of Cardiovascular Surgery of the General Hospital of Northern Theater Command between 1 January 2020 and 31 May 2020. Exclusion criteria were patients with chronic bowel disorders, long-term antibiotic therapy or Crohn’s disease and a history of arrhythmia, including AF and ventricular tachycardia (according to 7-day Holter). All patients underwent off-pump CABG. A total of 45 POAF patients and 90 matched controls (1:2) were enrolled. POAF was defined as any atrial fibrillation episode lasting ≥30 s after surgery, as detected with a 7-day Holter monitor equipped with an automated algorithm for detection of cardiac arrhythmia events (Yueguang Medical Technologies, Shanghai, China) AF events were confirmed by 2 physicians. Continuous telemetry and 7-day Holter monitoring were started after the surgery [1].

### 2.2. DNA Isolation, 16S rRNA Gene Amplification and Bioinformatics

Bacterial gDNA was extracted from fecal samples using the TIANamp Stool DNA Kit (TIANGEN, Shanghai, China) according to the manufacturer’s instructions. V3-V4, regions of the bacterial 16S ribosomal RNA gene, were amplified by PCR (16S V4: 515F-806R primers were used). All PCR reactions were performed with Phusion^®^ High-Fidelity PCR Master Mix (New England Biolabs, Ipswich, MA, USA). Samples with a bright main strip between 400 and 450 bp (16S) were chosen for further experiments. The PCR product mixture was purified with a Qiagen Gel Extraction Kit (Qiagen, CA, USA). After purification, the amplicon library was paired-end-sequenced (2 × 250) on an Illumina Hiseq platform (Beijing Capitalbio Technology, Beijing, China) according to the standard protocols [20]. Raw reads were filtered to remove adaptors and low-quality and ambiguous bases, and paired-end reads were added to tags by FLASH (version 1.2.11) [21]. Paired-end reads were assigned to each sample according to their unique barcodes. Sequences were analyzed using the QIIME (version 1.9.1) software package (Quantitative Insights Into Microbial Ecology) [22]. Operational taxonomic units (OTUs) were clustered with 97% similarity cutoff using UPARSE (version 11.0 http://drive5.com/uparse/ accessed on 1 October 2020), and chimeric sequences were identified and removed using UCHIME. The Chao richness and Shannon index were calculated at the genera levels using QIIME. Bray–Curtis dissimilarity β-diversity distances were generated after applying the negative binomial variance, stabilizing transformation using R packages vegan 2.5–5. Bray–Curtis-based principal coordinate analysis (PCoA) was conducted on ‰ OTU relative abundance log_2_-transformed using the R package phyloseq with the function ‘ordinate’. Principal component analysis (PCA) plots were generated based on the ‰ OTU relative abundance log_2_ transformed using the R package vegan 2.5–5. Microbiota-based biomarkers were identified with linear discriminant analysis effect size (LEfSe) using the online galaxy server (https://huttenhower.sph.harvard.edu/galaxy/ accessed on 1 October 2020), and the LDA scores derived from LEfSe analysis were used to demonstrate the relationship between taxon using a cladogram (circular hierarchical tree) of significantly increased or decreased bacterial taxa in the microbiota between groups [20].

### 2.3. Plasma Vitamin D Measurement

Plasma vitamin D levels were measured in duplicate using a commercially available enzyme-linked immunosorbent assay kit (Human 25-hydroxy Vitamin D, Downers Grove, IL, USA).

### 2.4. Systemic Inflammation

All serum inflammatory factors, including interleukin-6 (IL-6) and C-reactive protein (CRP), were quantified with Luminex assay technology (R&D Systems, Minneapolis, MN, USA).

### 2.5. Statistical Analysis

To balance the impact of treatment selection bias and potential confounders, we enrolled the patients in a 1:2 ratio from the POAF and no-POAF groups, and adjusted the differences in the patients’ baseline clinical characteristics using propensity score matching (PSM) using the optimal method of the MatchIt package in R software version 3.6.2. The selection process used a *p* value cutoff of 0.05 for entering and retaining data in the model [23].

The basic data were statistically analyzed using SPSS software version 24.0 (IBM SPSS Statistics, IBM Corporation, Armonk, NY, USA) and R software version 3.6.2 (R Foundation for Statistical Computing, Vienna, Austria). Continuous variables are described as medians (first, third quartile) and were evaluated using the Mann–Whitney U test. Categorical variables were compared using χ2 tests [24]. The Kruskal–Wallis H test was used in the comparison of the Shannon index and Chao1 index. In the comparison of PCoA analysis, an Adonis test was used. *p* < 0.05 was considered significant in all comparisons.

## 3. Results

### 3.1. Clinical Characteristics of Patients

A total of 150 consecutive patients successfully underwent off-pump CABG at the Department of Cardiovascular Surgery of the General Hospital of Northern Theater Command between 1 January 2020 and 31 May 2020. According to continuous telemetry and 7-day Holter monitoring, 45 patients developed POAF after the surgery. The 45 patients were selected as case subjects, and 90 patients (1:2) in the same cohort who did not develop POAF were selected as control subjects with matched characteristics (i.e., age, gender, body mass index, size of left atrium and ventricle, cardiac function and use of medicine before surgery). The clinical characteristics are represented in Table 1. One sample in the no-POAF group was deleted owing to not reaching plateaus for depth sequencing. There was no significant difference in clinical characteristics, cardiac function or serum biochemical parameters between the two groups (Table 1).

### 3.2. Diversity of the Fecal Microbiota in POAF Patients

**Figure 1:**
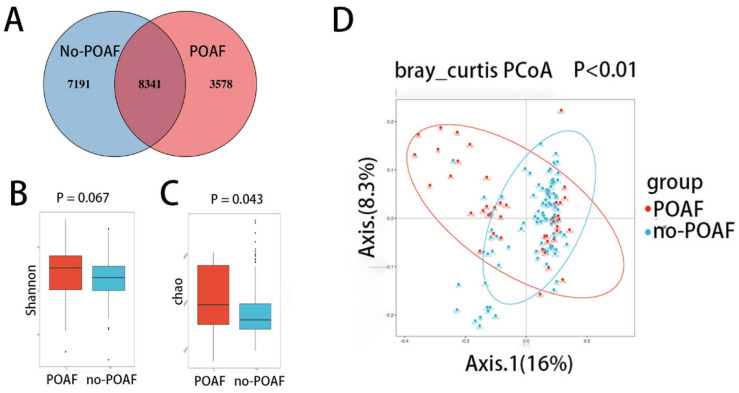
The diversity of the fecal microbiota. (**A**) OTU Venn diagram: blue circle indicates the number of OTUs unique to no-POAF patients, red circle indicates the number of OTUs unique to POAF, and the overlapping part shows the number of OTUs shared by the two groups. (**B**) The Shannon index in POAF and non-POAF patients. (**C**) The Chao1 index in POAF and non-POAF patients. (**D**) The β diversity of POAF and non-POAF patients based on the PCoA analysis. Samples were used in each analysis. Kruskal–Wallis H test was used in the comparison of the Shannon index and Chao1 index. In the comparison of PCoA analysis, the Adonis test was used.

The gut microbiota were evaluated using 16S rRNA sequencing in 45 POAF patients and matched 89 no-POAF patients. The two groups shared 8341 operational taxonomic units (OTUs), with 3578 and 7191 OTUs unique to the POAF group and the no-POAF group, respectively (Figure 1A). To assess gut microbial diversity in POAF patients, the Shannon index and Chao1 index were used to assess the ɑ diversity of the microbiota, while principal coordinate analysis (PCoA) was used for the β diversity. The differences in Shannon index scores between the POAF and the no-POAF patients were not statistically significant (Figure 1B, *p* = 0.067). The difference in Chao1 index scores between the POAF and no-POAF patients was statistically significant (Figure 1C, *p* = 0.043). Unlike patients who did not develop POAF, the within-individual (alpha) diversity was higher in POAF patients. Regarding the β diversity, the PCoA and PCA analysis showed significant differences between POAF and no-POAF patients (Figure 1D, *p* < 0.01 and Figure S1). These results show a distinctive bacterial composition in the two groups.

### 3.3. Taxonomic Changes in Gut Microbiota

**Figure 2:**
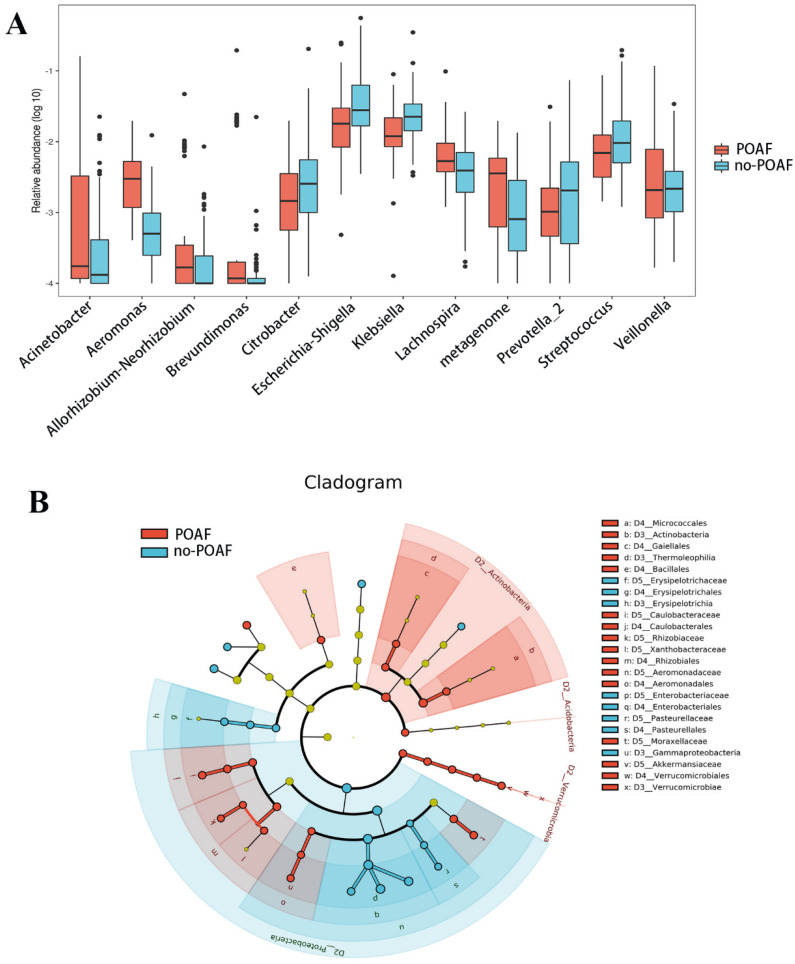
Differences in gut microbiota composition in patients with POAF and without POAF. (**A**) The box plots show the relative abundance of the genus between the two groups. Box, interquartile range; line inside a box, median; dot, outlier. (**B**) The results of linear discriminant analysis effect size (LEfSe) analysis.

Metastats was performed to further explore the distinguishing features between the POAF and no-POAF groups, and 21 distinct OTUs were found. At the genus level, the abundance of *Lachnospira* (*p* = 0.007), *Acinetobacter* (*p* < 0.001), *Veillonella* (*p* = 0.008) and *Aeromonas* (*p* < 0.001) was higher in POAF patients, while the abundance of *Escherichia–Shigella* (*p* = 0.043), *Klebsiella* (*p* = 0.002), *Streptococcus* (*p* = 0.029), *Brevundimonas* (*p* < 0.001) and *Citrobacter* (0.001) was significantly lower in POAF patients (Figure 2A and Supplementary Table S1).

Linear discriminant analysis (LDA) effect size (LEfSe) modeling was also applied to identify specific bacterial taxa associated with POAF (Figure 2B and Table S2). There were significant differences in community composition between the two groups. In the POAF group, the most abundant genera were *Actinobacteria* (LDA = 3.81, *p* < 0.001), *Caulobacterales* (LDA = 3.739, *p* = 0.027), *Brevundimonas* (LDA = 3.69, *p* = 0.008), *Acinetobacter* (LDA = 3.60, *p* = 0.020) and *Lachnospira* (LDA = 3.28, *p* = 0.008).

### 3.4. Correlations between Lachnospira and Plasma Vitamin D Levels

**Figure 3:**
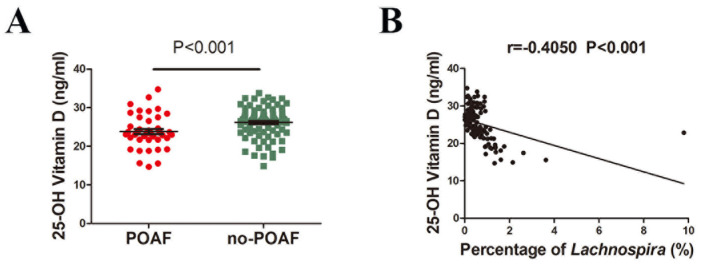
Correlations between Lachnospira and plasma Vitamin D levels. (**A**) Lower plasma 25-hydroxy vitamin D levels were found in the POAF group than the no-POAF group. *p* < 0.001 by unpaired *t* test. (**B**) Negative correlation between the plasma 25-hydroxy vitamin D levels and Lachnospira (r = −0.4050, *p* < 0.001 by Pearson correlation).

Given the association between *Lachnospira* and vitamin D intake [25], we compared the plasma 25-hydroxy vitamin D levels between patients with and without POAF. We found that 25-hydroxy vitamin D levels were lower in POAF patients compared to no-POAF patients (Figure 3A). Additionally, plasma 25-hydroxy vitamin D levels were negatively correlated with *Lachnospira* (r = −0.4050, *p* < 0.001) (Figure 3B).

## 4. Discussion

The present study showed new evidence for the characteristics of dysbiotic gut microbiota in patients who developed POAF after undergoing isolated CABG. We also found decreased plasma 25-hydroxy vitamin D levels in patients with POAF, which was negatively associated with the abundance of *Lachnospira*. These findings are fundamental for further studies exploring the precise mechanism of POAF.

This study confirmed that the structural characteristics of the gut microbiota were altered in patients who developed POAF compared to no-POAF patients. The difference in the α and β diversities of the POAF patients suggests that the richness and diversity of the microbiota had a significant difference between POAF patients and no-POAF patients. Previously, it has been shown that dysbiotic gut microbiota and alterations in metabolic patterns are involved in AF [26]. However, paroxysmal AF and persistent AF patients shared similarities and disparities in their gut microbiota profiles [13]. POAF is an appropriate model for studying the initiation of AF. Our findings, along with previous studies, indicate that the gut microbial composition had already shifted before AF. The alteration in gut microbiota may facilitate the initiation of AF. Our most recent study showed that administration of oral berberine, a common traditional Chinese medicine used for the treatment of diarrhea, before surgery could reduce the occurrence of POAF after CABG by decreasing the lipopolysaccharide levels after the surgery [27]. Cardiac surgery causes intestinal epithelial barrier dysfunction in patients who undergo CABG. Therefore, gut microbial composition before surgery may affect inflammation or oxidative stress in patients after surgery, which facilitates POAF.

According to the Metastats analysis, at the genus level, *Escherichia–Shigella* had the highest abundance of significantly altered gut microbiota. Compared with the no-POAF patients, patients with POAF harbored a nearly 50% abundance of *Escherichia–Shigella*. In a cohort of hypertension, the abundance of *Escherichia–Shigella* was significantly increased in cognition-impaired patients through inflammation-related mechanisms [28]. Moreover, another study indicated that *Escherichia–Shigella* was more abundant in decompensated heart failure than in compensated heart failure [29]. Nevertheless, the present study suggested that patients who developed POAF harbored low abundance of *Escherichia–Shigella*. Furthermore, this finding suggested further functional studies should be performed in the future.

Among the significantly increased gut microbiota, *Lachnospira* had the highest abundance. Our results indicate that patients who developed POAF showed nearly twice the abundance of *Lachnospira* compared to patients without POAF. A Japanese study found that *Lachnospira* had a significantly negative correlation with protein, sodium, iron, vitamin D, vitamin B6 and vitamin B12 intake, by comparing the gut microbiome of monozygotic twins [25]. Vitamin D deficiency may facilitate the initiation and development of AF through proliferative and pro-inflammatory actions of the renin–angiotensin system and excess catecholamine, interstitial fibrosis in the left atrium, and various electrical anomalies that cause fibrillatory conductions [30]. Left atrium fibrosis was reported to be negatively correlated with serum 25 (OH)D levels [31]. Many studies have demonstrated that interstitial fibrosis increases AF susceptibility [32,33,34,35]. Furthermore, serum vitamin D deficiency is associated with an increased risk of POAF development [36,37]. Meanwhile, vitamin D supplementation reduces the incidence of POAF in vitamin D insufficiency patients [38,39,40]. This study provided a possible mechanism of vitamin D prevention in POAF through altering the abundance of *Lachnospira.* However, another study demonstrated that *Lachnospira* were increased in most AF patients after catheter ablation. In line with the previous findings, this study showed that plasma 25-hydroxy vitamin D levels were negatively associated with *Lachnospira*, indicating that altering the abundance of *Lachnospira* may represent a new possible POAF treatment.

## 5. Limitations

First, this study was performed in a Chinese population whose diet mainly consists of cereals and cereal products. Since gut microbiota are critically determined by diet, the Chinese diet may limit the generalizability of our findings. Second, 16S amplicon sequencing provides limited taxonomic and is inadequate for functional analysis, and microbiome metagenomics should be performed in the future [28]. Thirdly, the small group size in this study makes it possible to ignore slight alterations in the gut microbiota.

## 6. Conclusions

The present study indicates that the gut microbiota composition between patients with and without POAF is significantly different, implying that gut microbiota may play a role in the pathogenesis of POAF. Intervention strategies targeting dysbiotic gut microbiota to prevent POAF may be clinically valuable.

## Figures and Tables

**Table 1:** Demographic and clinical characteristics of the patients at baseline.

Characteristic	POAF (*n* = 45)	NO-POAF (*n* = 89)	*p*
Age (y)	64.6 (60, 69.50)	63.5 (59.75, 67.25)	0.495
Female sex, %	13 (28.88)	24 (26.96)	0.887
Body mass index	24.78 (22.4, 27.1)	25.17 (23.15, 26.65)	0.425
Smoking history, %	24 (53.33)	49 (55.05)	0.903
Hypertension, %	24 (53.33)	55 (61.79)	0.389
Diabetes mellitus, %	17 (37.78)	35 (39.3)	0.804
Hyperlipidemia, %	19 (42.22)	36 (40.45)	0.806
NYHA = I–III			
I	18 (40.00)	29 (32.22)	1.000
II	24 (53.33)	55 (61.79)	
III	3 (6.67)	5 (5.56)	
Left ventricular ejection fraction, %	57.1 (56, 60)	56.2 (53, 60)	0.459
Left atrial size (mm)	37.78 (34, 41)	36.76 (33.75, 39)	0.122
LVEDV (mL)	100.4 (89, 105)	101.44 (83.75, 112.50)	0.785
LMCA stenosis (≥50%)	14 (31.11)	27 (30.30)	0.895
Right coronary stenosis (≥70%)	36 (80.00)	73 (82.02)	0.878
Calcium channel blockers, %	25 (55.56)	52 (58.24)	0.806
Beta-blockers, %	45 (100)	89 (100)	1.000
Use statins, %	7 (15.56)	15 (16.85)	0.870
creatinine (mg/dL)	69.35 (54.9, 77.47)	73.80 (61.91, 85.43)	0.144
BNP (pg/mL)	447.72 (85.6, 427.25)	538.98 (108.47, 581.05)	0.444
CKMB	11.12 (7.60, 13.75)	11.90 (9.00, 14.00)	0.176
HsTnT (ng/mL)	0.173 (0.010, 0.059)	0.098 (0.01, 0.463)	0.567
CRP (mg/L)	2.80 (0.20, 29.20)	3.050 (0.20, 76.60)	0.758
IL-6 (pg/mL)	6.56 (1.85, 34.97)	5.36 (1.58, 70.81)	0.268

Values are medians (1st, 3rd quartile) or *n*; NYHA: New York Heart Association; LVEDV: left ventricular end-diastolic volume; LMCA: left main coronary artery; CRP: C-reactive protein; IL-6: Interleukin-6.

## Data Availability

The datasets presented in this study can be found in online repositories. The names of the repository/repositories and accession number(s) can be found below: https://www.ncbi.nlm.nih.gov/, PRJNA832570.

## Supplementary Materials

The following supporting information can be downloaded at: https://www.mdpi.com/article/10.3390/jcm12041493/s1, Table S1: Differences in gut microbiota composition in patients with POAF and no POAF. Table S2: Linear discriminant analysis (LDA) effect size (LEfSe) modeling and POAF. Figure S1: Systemic inflammation after 48 h of surgery.
