# Supplementary material for: Gut Microbiota in Patients with Postoperative Atrial Fibrillation Undergoing Off-Pump Coronary Bypass Graft Surgery
Source: J Clin Med. 2023 Feb 13;12(4):1493. doi: 10.3390/jcm12041493 (PMC9960524; doi:10.3390/jcm12041493)
Supplement: Supplementary file 1 [file jcm-12-01493-s001.zip › Table supplementary S1.pdf]

Supplementary Table S1. Differences in gut microbiota composition in patients with POAF and no POAF.

**Taxonomy**	**Mean (POAF)**	**Variance (POAF)**	**Std.err (PAOF)**	**Mean (no-POAF)**	**Variance (no_POAF)**	**Std.err (no_POAF)**	***p* Value**
Escherichia-Shigella	0.031794922	0.002655	0.007768	0.058859	0.007993	0.009424	0.043
Klebsiella	0.01844616	0.00032	0.002697	0.032741	0.001682	0.004323	0.002
Streptococcus	0.011741429	0.000223	0.002252	0.021138	0.001193	0.003641	0.029
Lachnospira	0.009078976	0.000225	0.002261	0.005029	2.09×10^{-5}	0.000482	0.007309
Brevundimonas	0.008099381	0.000888	0.004492	0.000283	5.46×10^{-6}	0.000246	1.02×10^{-14}
Acinetobacter	0.007484666	0.000612	0.00373	0.00107	1.03×10^{-5}	0.000338	4.30×10^{-9}
Veillonella	0.007247389	0.000324	0.002714	0.00378	3.19×10^{-5}	0.000595	0.00837
Aeromonas	0.003795764	1.49×10^{-5}	0.000582	0.000841	2.21×10^{-6}	0.000157	0.000382
Citrobacter	0.002899961	1.77×10^{-5}	0.000634	0.007358	0.000507	0.002373	0.001597
Prevotella_2	0.002642426	3.22×10^{-5}	0.000856	0.005454	0.000142	0.001256	0.028578
Allorhizobium-Neorhizobium-Pararhizobium-Rhizobium	0.002637651	5.74×10^{-5}	0.001142	0.000264	8.95×10^{-7}	9.97×10^{-5}	6.89×10^{-5}
Ruminococcus_gnavus_group	0.002379556	1.10×10^{-5}	0.000501	0.006893	0.000218	0.001557	0.000358
Microbacterium	0.002371896	5.72×10^{-5}	0.00114	0.000166	6.17×10^{-7}	8.28×10^{-5}	0.000112
Alloprevotella	0.002155109	3.82×10^{-5}	0.000932	0.007141	0.00019	0.001453	8.22×10^{-5}
Candidatus_Udaeobacter	0.001583488	2.98×10^{-6}	0.00026	0.00049	1.55×10^{-6}	0.000131	0.048068
Barnesiella ,	0.001474752	5.91×10^{-6}	0.000366	0.003245	6.70×10^{-5}	0.000863	0.048451
Pseudolabrys	0.001172537	1.84×10^{-6}	0.000204	0.000268	4.07×10^{-7}	6.73×10^{-5}	0.042423
Caulobacter	0.000861221	8.41×10^{-6}	0.000437	5.01×10^{-5}	7.19×10^{-8}	2.83×10^{-5}	0.011614

Leuconostoc	0.000670923	4.23×10^{-6}	0.00031	4.06×10^{-5}	4.39×10^{-8}	2.21×10^{-5}	0.035387
Haemophilus	0.000641004	8.86×10^{-7}	0.000142	0.002555	5.33×10^{-5}	0.00077	0.020424
Olsenella	0.000122325	1.35×10^{-7}	5.53×10^{-5}	0.002921	0.000459	0.002259	0.000628
