# Supplementary material for: Gut Microbiota in Patients with Postoperative Atrial Fibrillation Undergoing Off-Pump Coronary Bypass Graft Surgery
Source: J Clin Med. 2023 Feb 13;12(4):1493. doi: 10.3390/jcm12041493 (PMC9960524; doi:10.3390/jcm12041493)
Supplement: Supplementary file 1 [file jcm-12-01493-s001.zip › Table supplementary S2.pdf]

Supplementary Table S2.

Biomarkers	Groups	LDA	p
D1__Bacteria.D2__Proteobacteria.D3__Gammaproteobacteria.D4__Enterobacteriales	No-POAF	4.355336	0.000159
D1__Bacteria.D2__Proteobacteria.D3__Gammaproteobacteria.D4__Enterobacteriales.D5__Enterobacteriaceae	No-POAF	4.355336	0.000159
D1__Bacteria.D2__Proteobacteria.D3__Gammaproteobacteria	No-POAF	4.240385	0.001577
D1__Bacteria.D2__Proteobacteria.D3__Gammaproteobacteria.D4__Enterobacteriales.D5__Enterobacteriaceae.D6__Escherichia_Shigella	No-POAF	4.106985	0.001274
D1__Bacteria.D2__Proteobacteria	No-POAF	3.995073	0.022958
D1__Bacteria.D2__Actinobacteria	POAF	3.918087	7.18×10^{-5}
D1__Bacteria.D2__Proteobacteria.D3__Gammaproteobacteria.D4__Enterobacteriales.D5__Enterobacteriaceae.D6__Klebsiella	No-POAF	3.87509	0.000515
D1__Bacteria.D2__Actinobacteria.D3__Actinobacteria	POAF	3.806372	0.001339
D1__Bacteria.D2__Proteobacteria.D3__Alphaproteobacteria.D4__Caulobacterales	POAF	3.739217	0.027078
D1__Bacteria.D2__Proteobacteria.D3__Alphaproteobacteria.D4__Caulobacterales.D5__Caulobacteraceae	POAF	3.738413	0.027432
D1__Bacteria.D2__Proteobacteria.D3__Alphaproteobacteria.D4__Caulobacterales.D5__Caulobacteraceae.D6__Brevundimonas	POAF	3.690953	0.008309
D1__Bacteria.D2__Proteobacteria.D3__Gammaproteobacteria.D4__Pseudomonadales.D5__Moraxellaceae	POAF	3.632391	0.025798
D1__Bacteria.D2__Proteobacteria.D3__Gammaproteobacteria.D4__Pseudomonadales.D5__Moraxellaceae.D6__Acinetobacter	POAF	3.605187	0.020519
D1__Bacteria.D2__Proteobacteria.D3__Alphaproteobacteria.D4__Rhizobiales	POAF	3.557477	0.007893
D1__Bacteria.D2__Bacteroidetes.D3__Bacteroidia.D4__Bacteroidales.D5__Prevotellaceae.D6__Alloprevotella	No-POAF	3.424863	7.66×10^{-7}
D1__Bacteria.D2__Actinobacteria.D3__Actinobacteria.D4__Micrococcales	POAF	3.377931	0.018187
D1__Bacteria.D2__Firmicutes.D3__Clostridia.D4__Clostridiales.D5__Lachnospiraceae.D6__Ruminococcus_gnavus_group	No-POAF	3.347146	0.000837
D1__Bacteria.D2__Proteobacteria.D3__Gammaproteobacteria.D4__Enterobacteriales.D5__Enterobacteriaceae.D6__Citrobacter	No-POAF	3.330902	0.009554
D1__Bacteria.D2__Firmicutes.D3__Clostridia.D4__Clostridiales.D5__Ruminococcaceae.D6__Ruminococcus_2	No-POAF	3.321532	0.007863
D1__Bacteria.D2__Actinobacteria.D3__Thermoleophilia	POAF	3.312396	0.010172

D1__Bacteria.D2__Firmicutes.D3__Clostridia.D4__Clostridiales.D5__Lachnospiraceae.D6__Lachnospira	POAF	3.279777	0.008672
D1__Bacteria.D2__Actinobacteria.D3__Coriobacteriia.D4__Coriobacteriales.D5__Atopobiaceae.D6__Olsenella	No-POAF	3.253476	0.016656
D1__Bacteria.D2__Firmicutes.D3__Erysipelotrichia.D4__Erysipelotrichales	No-POAF	3.224411	0.026929
D1__Bacteria.D2__Firmicutes.D3__Erysipelotrichia	No-POAF	3.224411	0.026929
D1__Bacteria.D2__Firmicutes.D3__Erysipelotrichia.D4__Erysipelotrichales.D5__Erysipelotrichaceae	No-POAF	3.224411	0.026929
D1__Bacteria.D2__Verrucomicrobia.D3__Verrucomicrobiae	POAF	3.220105	0.002466
D1__Bacteria.D2__Verrucomicrobia	POAF	3.220105	0.002466
D1__Bacteria.D2__Actinobacteria.D3__Thermoleophilia.D4__Gaiellales	POAF	3.206438	0.015882
D1__Bacteria.D2__Proteobacteria.D3__Gammaproteobacteria.D4__Aeromonadales	POAF	3.203835	2.05×10^{-12}
D1__Bacteria.D2__Proteobacteria.D3__Gammaproteobacteria.D4__Aeromonadales.D5__Aeromonadaceae	POAF	3.180808	3.64×10^{-12}
D1__Bacteria.D2__Proteobacteria.D3__Gammaproteobacteria.D4__Aeromonadales.D5__Aeromonadaceae.D6__Aeromonas	POAF	3.180808	3.64×10^{-12}
D1__Bacteria.D2__Acidobacteria	POAF	3.179249	0.025204
D1__Bacteria.D2__Proteobacteria.D3__Alphaproteobacteria.D4__Rhizobiales.D5__Rhizobiaceae	POAF	3.171325	0.025312
D1__Bacteria.D2__Proteobacteria.D3__Alphaproteobacteria.D4__Rhizobiales.D5__Rhizobiaceae.D6__Allorhizobium_Neorhizobium_Pararhizobium_Rhizobium	POAF	3.164891	0.02613
D1__Bacteria.D2__Firmicutes.D3__Bacilli.D4__Bacillales	POAF	3.146562	0.031855
D1__Bacteria.D2__Verrucomicrobia.D3__Verrucomicrobiae.D4__Verrucomicrobiales	POAF	3.139919	0.013935
D1__Bacteria.D2__Verrucomicrobia.D3__Verrucomicrobiae.D4__Verrucomicrobiales.D5__Akkermansiaceae	POAF	3.139916	0.010514
D1__Bacteria.D2__Verrucomicrobia.D3__Verrucomicrobiae.D4__Verrucomicrobiales.D5__Akkermansiaceae.D6__Akkermansia	POAF	3.139916	0.010514
D1__Bacteria.D2__Proteobacteria.D3__Alphaproteobacteria.D4__Rhizobiales.D5__Xanthobacteraceae	POAF	3.111256	0.004547
D1__Bacteria.D2__Proteobacteria.D3__Gammaproteobacteria.D4__Pasteurellales.D5__Pasteurellaceae	No-POAF	3.055787	7.77×10^{-5}
D1__Bacteria.D2__Proteobacteria.D3__Gammaproteobacteria.D4__Pasteurellales	No-POAF	3.055787	7.77×10^{-5}

D1__Bacteria.D2__Proteobacteria.D3__Gammaproteobacteria. D4__Pasteurellales.D5__Pasteurellaceae.D6__Haemophilus	No-POAF	3.022902	9.46×10^{-5}
